# Impact of Trail Running Races on Blood Viscosity and Its Determinants: Effects of Distance

**DOI:** 10.3390/ijms21228531

**Published:** 2020-11-12

**Authors:** Mélanie Robert, Emeric Stauffer, Elie Nader, Sarah Skinner, Camille Boisson, Agnes Cibiel, Léonard Feasson, Céline Renoux, Paul Robach, Philippe Joly, Guillaume Y. Millet, Philippe Connes

**Affiliations:** 1 Laboratoire Interuniversitaire de Biologie de la Motricité (LIBM) EA7424, Team “Vascular Biology and Red Blood Cell”, Université Claude Bernard Lyon 1, Université de Lyon, Lyon, France; melanie.robert@erytech.com (M.R.); emeric.stauffer@chu-lyon.fr (E.S.); elie.nader@free.fr (E.N.); scs9yh@virginia.edu (S.S.); camille.boisson2@gmail.com (C.B.); celine.renoux@chu-lyon.fr (C.R.); philippe.joly@chu-lyon.fr (P.J.); 2 Laboratoire d’Excellence du Globule Rouge (Labex GR-Ex), PRES Sorbonne, 75015 Paris, France; 3 Erytech Pharma, 69008 Lyon, France; agnes.cibiel@erytech.com; 4 Centre de Médecine du Sommeil et des Maladies Respiratoires, Hôpital Croix Rousse, Hospices Civils de Lyon, Lyon, France; 5 Laboratoire Interuniversitaire de Biologie de la Motricité, UJM-Saint-Etienne, Univ Lyon, EA 7424, F-42023, Saint-Etienne, France; leonard.feasson@univ-st-etienne.fr (L.F.); guillaume.millet@univ-st-etienne.fr (G.Y.M.); 6 Unité de Myologie, Service de Physiologie Clinique et de l’Exercice, Hôpital Universitaire de Saint-Etienne, Saint-Etienne, France; 7 Laboratoire de Biochimie et de Biologie Moléculaire, UF de Biochimie des Pathologies Érythrocytaires, Centre de Biologie et de Pathologie Est, Hospices Civils de Lyon, Lyon, France; 8 National School for Mountain Sports, Site of the National School for Skiing and Mountaineering, Chamonix, France; paul.robach@ensm.sports.gouv.fr; 9 Institut Universitaire de France (IUF), Paris, France

**Keywords:** ultra-marathon, hemorheology, red blood cell senescence, blood viscosity

## Abstract

Blood rheology is a key determinant of tissue perfusion at rest and during exercise. The present study investigated the effects of race distance on hematological, blood rheological, and red blood cell (RBC) senescence parameters. Eleven runners participated in the Martigny–Combes à Chamonix 40 km race (MCC, elevation gain: 2300 m) and 12 others in the Ultra-Trail du Mont Blanc (UTMB, 171 km, elevation gain: 10,000 m). Blood samples were collected before and after the races. After the UTMB, the percentage of RBC phosphatidylserine (PS) exposure was not affected while RBC CD235a levels decreased and RBC-derived microparticles increased. In contrast, after the MCC, RBC PS exposure increased, while RBC CD235a and RBC-derived microparticles levels were not affected. The free hemoglobin and hemolysis rate did not change during the races. RBC aggregation and blood viscosity at moderate shear rates increased after the MCC. RBC deformability, blood viscosity at a high shear rate, and hematocrit decreased after the UTMB but not after the MCC. Our results indicate that blood rheology behavior is different between a 40 km and a 171 km mountain race. The low blood viscosity after the ultra-marathon might facilitate blood flow to the muscles and optimize aerobic performance.

## 1. Introduction

Red blood cells (RBC) need to be highly deformable to flow through the microcirculation and efficiently deliver their oxygen to the surrounding tissues [1,2,3]. Indeed, any decrease in RBC deformability may affect oxygen delivery and exercise performances [4,5]. Moreover, any decrease in RBC deformability may cause a rise in blood viscosity [1], which may increase the vascular resistance and the cardiovascular strain, as well as impair local blood flow [6]. RBCs are also able to form reversible aggregates. Enhanced RBC aggregation increases blood viscosity, which in turn may affect blood flow [7,8].

Previous studies have shown that RBC integrity and deformability are well preserved during maximal or submaximal running efforts ranging from less than 15 min to more than ~3 h in duration [9,10,11]. RBC aggregation was also unaffected by a maximal exercise running test [12], a 10 km [11], or a marathon [10]. Finally, blood viscosity was not affected by a short maximal exercise test conducted in laboratory conditions [12], a marathon [10], or a 10-km bout of running performed in either a tropical climate [11] or a neutral environment [9]. 

Long-distance running has become increasingly popular, but the literature on the impact of such exercise on RBC rheology and integrity, as well as on blood viscosity, is limited. Systemic oxidative stress and inflammation are highly increased during an ultra-marathon [13,14,15]. Elevated oxidative stress and enhanced inflammation may cause RBC damage and promote RBC senescence, characterized by, among other alterations, cell shrinkage, a loss of RBC deformability, membrane vesiculation, and increased phosphatidylserine (PS) exposure on the outer leaflet of the RBC membrane [16]. Indeed, prolonged running efforts, in contrast to shorter ones [9], may result in RBC damage and a loss in RBC deformability. However, because plasma volume expansion occurs during ultra-trails [17], one could speculate that the subsequent decrease in hematocrit will compensate for the changes in RBC rheology, resulting in a lack of change or a decrease in blood viscosity in comparison to shorter running efforts. 

This study aimed to compare the effects of running distance, i.e., a ‘classic’ trail running race vs. an ultra-trail, on RBC rheological properties and markers of RBC damage, as well as blood viscosity. Our results showed that blood rheology parameters and markers of RBC damage are modulated differently after a 40 km or 171 km mountain race; the longer effort resulted in (1) decreased RBC deformability accompanied by an emission of RBC-related microparticles (MPs) and (2) a significant reduction in hematocrit and blood viscosity, without any sign of increased hemolysis. 

## 2. Results

### 2.1. Subjects Characteristics

Eleven runners participated in the “Martigny–Combes à Chamonix” race (MCC, 40 km, elevation gain: 2300 m), and 12 runners participated in the “Ultra-Trail du Mont Blanc” race (UTMB^©^, 171 km, elevation gain: 10,000 m). MCC and UTMB participants had similar maximal oxygen uptake (VO_2_max; 59.0 ± 11.6 and 59.7 ± 7.2 mL/min/kg, respectively). Mean race time was 6 h 50 min and 39 h 53 min for the MCC and UTMB runners, respectively. Average running speed was higher during the MCC (6.1 ± 1.6 km/h) than during the UTMB (4.4 ± 0.9 km/h) race (*p* < 0.01). The two groups lost weight after the race (before/after-race: from 65.4 ± 10.2 kg to 63.6 ± 9.8 kg, *p* < 0.001, and from 63.3 ± 8.9 kg to 62.5 ± 8.7 kg, *p* < 0.05, for MCC and UTMB participants, respectively) but weight loss was higher in the MCC (−2.8 ± 1.0%) than in the UTMB (−1.2 ± 1.8%) runners (*p* < 0.01). Gender repartition was not different between the two groups (chi^2^: 1.051). 

### 2.2. UTMB Caused a Decrease in Hematocrit, RBC Deformability, and Blood Viscosity, While MCC Increased Blood Viscosity 

**Figure 1:**
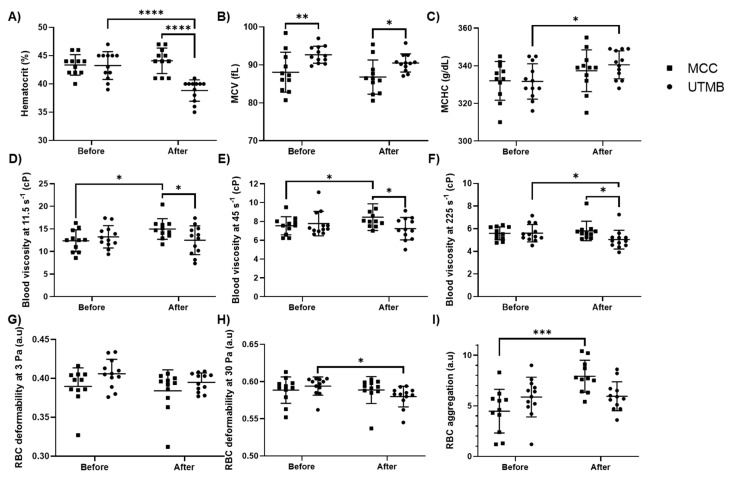
Evolution of hematological and hemorheological parameters before and after Ultra-Trail du Mont Blanc (UTMB) (*n* = 12) or Martigny–Combes à Chamonix (MCC) (*n* = 11) race. (**A**) Hematocrit (Hct); (**B**) Mean corpuscular volume (MCV); (**C**) Mean corpuscular hemoglobin concentration (MCHC); Blood viscosity measured at (**D**) 11.5 s^−1^ (**E**) 45 s^−1^, (**F**) 225 s^−1^; red blood cell (RBC) deformability measured at (**G**) 3 Pa, (**H**) 30 Pa and (**I**) RBC aggregation. Values are represented as means ± SD. Statistical difference: * *p* < 0.05, ** *p* < 0.01, *** *p* < 0.001, **** *p* < 0.0001.

Hematological parameters were assessed before and after each race. Hematocrit (Hct) did not change in MCC participants (from 43.4 ± 1.9% to 44.1 ± 2.2%), while it decreased in UTMB runners (from 43.3 ± 2.5% to 38.8 ± 1.9%; *p* < 0.001, Figure 1A). Hct was lower in UTMB than in MCC after the race (*p* < 0.001, Figure 1A). The mean corpuscular volume (MCV) was slightly but significantly different between the two groups before (*p* < 0.01) and after the race (*p* < 0.05), but the exercise had no impact on this parameter (Figure 1B). RBC width distribution (RDW) was not different between the two groups and was not affected by the races (data not shown).

The mean corpuscular hemoglobin concentration (MCHC) was not significantly affected by the MCC but increased in UTMB runners after the race (Figure 1C). Next, we evaluated the hemorheological parameters. Blood viscosity measured at 11.5 and 45 s^−1^ increased in MCC runners after the race (*p* < 0.05), while it remained unchanged in UTMB subjects (Figure 1D,E). At 225 s^−1^, blood viscosity was not affected by the MCC but decreased in UTMB runners after the race compared to pre-exercise (*p* < 0.05, Figure 1F). MCC subjects had higher blood viscosity (at the three shear rates) than UTMB subjects after the race (*p* < 0.05). No significant change in RBC deformability was observed at 3 Pa, but a decrease was observed at 30 Pa in the UTMB group only (Figure 1G,H). RBC aggregation increased after the race in MCC runners (*p* < 0.001) but not in the UTMB group (Figure 1I).

### 2.3. UTMB Promoted the Release of RBC Microparticles without Any Sign of Hemolysis, While MCC Promoted RBC Senescence without Any Increase in RBC Microparticles

**Figure 2:**
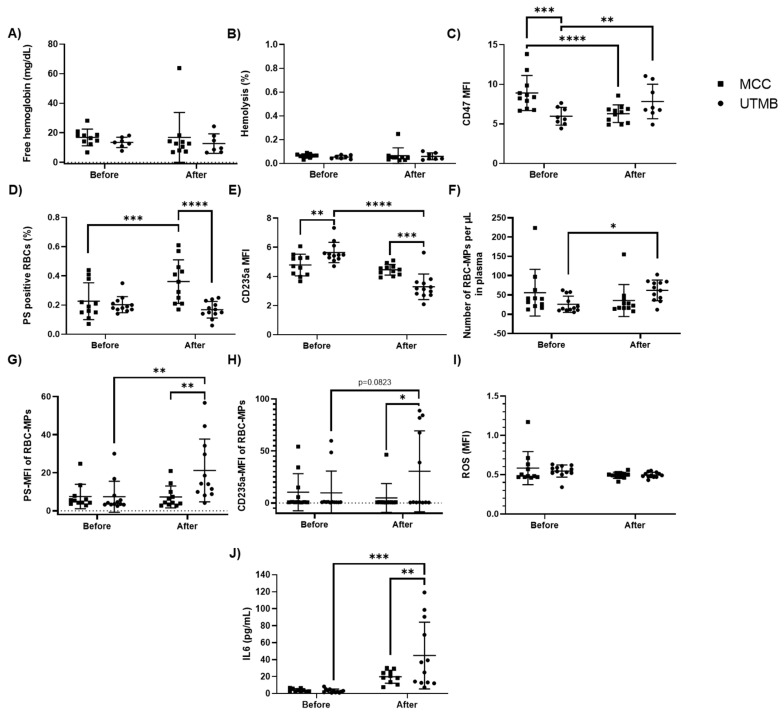
Evolution of markers of RBC damage and senescence and IL6 levels before and after UTMB or MCC race. (**A**) Plasma free hemoglobin (UTMB, *n* = 7; MCC, *n* = 10); (**B**) Percentage of hemolysis (UTMB, *n* = 7; MCC, *n* = 10); (**C**) Mean fluorescence intensity (MFI) of CD47 expressed at RBC surface (UTMB, *n* = 8; MCC, *n* = 11); (**D**) Percentage of RBC exposing phosphatidylserine (PS); (**E**) MFI of CD235a expressed at RBC surface; (**F**) Number of RBC-microparticles (MPs) per µL in plasma; (**G**) Mean fluorescence intensity of PS expressed in RBC-MPs; (**H**) MFI of CD235a expressed in RBC-MPs; (**I**) RBC Reactive oxygen species; (**J**) Plasma interleukin 6 (IL-6) content. When not specified 12 samples were analyzed for the UTMB and 11 samples for the MCC. Values are represented as mean ± SD. Statistical difference: * *p* < 0.05, ** *p* < 0.01, *** *p* < 0.001, **** *p* < 0.0001.

We then investigated the impact of MCC and UTMB on markers of RBC damage and senescence. The level of plasma free hemoglobin did not differ between the two groups and was not affected by the exercise (Figure 2A). The low levels of free hemoglobin detected in plasma indicated that there was a low percentage of hemolysis (<0.2%), with similar levels before and after exercise and between the two races (Figure 2B). Using flow-cytometry, we determined the levels of CD47 (a marker of self on RBC), glycophorin A (CD235a), and the percentage of PS exposure on the RBC surface. The mean fluorescence intensity (MFI) of RBC-CD47 decreased after the race in MCC individuals (*p* < 0.05), while it surprisingly increased in the UTMB group (*p* < 0.001, Figure 2C). RBC-CD47 MFI was higher in MCC runners compared to UTMB runners before the race (*p* < 0.01), but was lower after the race (*p* < 0.001, Figure 2C). PS exposure at the surface of RBC increased in MCC runners after the race (*p* < 0.01) and was higher than the UTMB runners post-race (*p* < 0.001, Figure 2D). The UTMB had no impact on RBC-PS exposure (Figure 2D). The MFI of RBC-CD235a decreased after the UTMB (*p* < 0.0001), while no change was observed after the MCC (Figure 2E). The level of RBC-CD235 MFI was higher in UTMB runners compared to MCC runners before the race (*p* < 0.01) and lower after the race (*p* < 0.001, Figure 2E). Next, we evaluated RBC membrane vesiculation after the races by assessing the levels of MPs released by RBC (RBC-MPs). Low levels of RBC-MPs were observed for the two races, but the UTMB caused a release of RBC-MPs into the plasma (*p* < 0.05), while the MCC had no effect on this parameter (Figure 2F). The MFI of PS exposed at the RBC-MPs surface was increased after the UTMB (*p* <0.01). Similar trends were observed for the MFI of RBC-MPs-CD235a (*p* = 0.0823). In contrast, the MFI of both RBC-MPs-PS and -CD235a remained unaffected by the MCC (Figure 2G,H). Indeed, the MFI levels of PS (*p* < 0.01) and CD235a (*p* < 0.05) of RBC-MPs were higher in UTMB runners compared to MCC runners after the race (Figure 2G,H). To determine the involvement of oxidative stress in triggering senescence markers, we evaluated the intracellular levels of reactive oxygen species (ROS) in the RBC. RBC-ROS content did not differ between the two groups and was not affected by the races (Figure 2I). Finally, we measured interleukin 6 (IL-6) to assess the level of inflammation. The MCC did not cause a significant change in plasma IL-6 level (*p* = 0.1), while the UTMB caused a large increase (*p* < 0.001, Figure 2J). At the end of the race, UTMB runners had higher IL-6 levels than MCC runners (*p* < 0.01, Figure 2J).

### 2.4. Correlations Between the Changes in Hematocrit and Weight Loss or IL6 Variations

**Figure 3:**
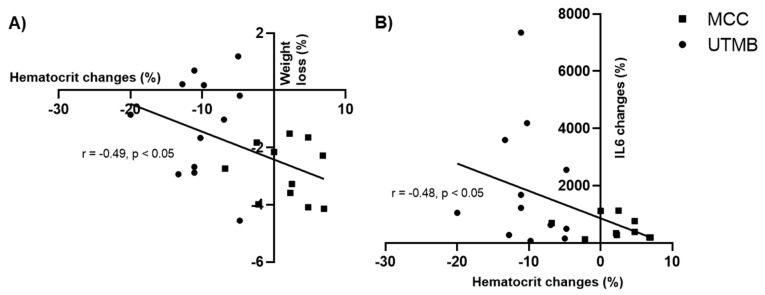
Correlations between the changes in hematocrit observed in both races and (**A**) weight loss; (**B**) IL-6 changes. All changes are expressed in percent of the resting values.

Percent of changes between pre- and post-race was calculated for several parameters for all of the 23 runners. The changes in Hct were significantly correlated with the corresponding changes in weight loss (−0.49; *p* < 0.05; Figure 3A) and IL-6 level (−0.48; *p* < 0.05; Figure 3B). These correlations were not observed in each group analyzed separately. No significant correlation was observed between the magnitude of change in Hct and the percent of changes in RBC deformability (r = 0.14; *p* = 0.53). In addition, the extent of hemolysis did not correlate with the percent of changes in RBC deformability (at 3Pa: r = 0.20; *p* = 0.42; at 30 Pa: r = 0.08; *p* = 0.75).

## 3. Discussion

The present study showed that (1) neither the MCC nor the UTMB, resulted in marked hemolysis. However, runners who finished the UTMB had a slight decrease in RBC deformability with increased RBC-MP levels, whereas runners who completed the MCC exhibited signs of RBC senescence without any significant changes in RBC deformability or MPs. (2) Blood viscosity increased at moderate shear rates in runners who completed the MCC, while it decreased at high shear rates in those who ran the UTMB. (3) RBC aggregation increased after the MCC but not after the UTMB. 

Previous studies have suggested that foot-strike hemolysis may occur during running events [18]. For example, Robach et al. [17] reported a significant, although moderate, decrease in serum haptoglobin after a 166-km long mountain ultra-endurance marathon with 9500 m of altitude gain/loss, suggesting that hemolysis occurred during the race. Free hemoglobin did not change after the MCC or after the UTMB in our study. In addition, the significant decrease observed in RBC deformability after the UTMB was very slight (−2.4%), indicating that RBCs were probably not severely damaged during the races. Furthermore, the magnitude of the reduction in RBC deformability did not correlate with the change in plasma free hemoglobin levels. 

RBC deformability depends on several factors, including the internal viscosity, the surface to volume ratio, and the viscoelastic properties of the membrane of the RBCs [1]. Indeed, the slight decrease in RBC deformability observed after the UTMB could be attributed to the slight increase in MCHC. Although this change could suggest that RBC dehydration occurred, the decrease in MCV did not reach statistical significance, and RDW did not change. In contrast, the release of MPs by RBCs during the UTMB could be responsible for a decrease in RBC surface area, which would cause the RBC surface/volume ratio to decrease, resulting in reduced RBC deformability [19]. 

While we previously demonstrated that a 10 km race had no impact on RBC senescence markers (PS, CD47, MPs, ROS) [9], the present study showed that ultra-endurance running affects these markers. The effects of the UTMB and MCC on RBC senescence markers were different. Oxidative stress is a key trigger of RBC senescence [16]. However, the lack of change in RBC-ROS levels during the two races suggests that oxidative stress was not involved in the modulation of RBC senescence markers in the present study. This result is supported by the lack of change in the reduced and oxidized glutathione ratio (GSH:GSSG ratio, data not shown). Increased RBC PS exposure after the MCC could have been caused by a rise in intracellular calcium, which would have led to a rupture of membrane phospholipid asymmetry [20]. However, one may note that the percent of PS exposed at the RBC membrane surface after the MCC remained rather low, as compared to other situations where PS exposure may increase over 1–2%, as it is the case in some hematological diseases or in blood bags before transfusion [21,22,23]. Among the triggers of RBC-MP production, Burger et al. [24] described that potassium leakage (through the activation of Gardos channels) could cause membrane disturbances leading to PS exposure and MP generation. Furthermore, inflammation is known to affect the RBC, as various cytokines can activate Gardos channels and cause intracellular water loss. In our study, plasma IL-6 levels significantly increased in UTMB runners, indicating that there was likely an enhanced pro-inflammatory response in this group that could have affected RBCs. Interestingly, Burger et al. [24] also described that RBCs can return to a PS-negative state by releasing MPs containing PS positive membranes. This may explain why the PS level of RBC-MPs increased after the UTMB, while the percentage of PS exposure at the RBC surface did not change. MPs release after the UTMB could limit senescence mechanisms. In contrast, MPs were not released by RBCs after the MCC. Therefore, senescence markers accumulated at the RBC membrane level (i.e., increased PS exposure). Our findings also showed that CD47 expression decreased after the MCC. This finding suggests advanced RBC senescence after the 40 km race as CD47 expression constitutes a “don’t eat me” signal [25,26]. In contrast, CD47 expression increased following the UTMB. Further studies are needed to understand why CD47 expression could increase after an ultramarathon.

One of the most intriguing findings of the present study was the different effects of the two races on blood viscosity. Blood viscosity increased in MCC runners at moderate shear rates, while it remained unchanged in the UTMB runners. Blood viscosity at low/moderate shear rates is highly dependent on hematocrit and RBC aggregation [1]. Although there were no significant changes in hematocrit following the MCC, RBC aggregation increased two-fold. Therefore, the elevated blood viscosity at moderate shear rates could have been caused by the increased RBC aggregation observed after the MCC. RBC aggregation depends on both plasma and cellular (RBC aggregability) factors, and older RBC exhibit higher RBC aggregability than younger RBCs [27]. We observed increased RBC-PS exposure after the MCC, which indicates that eryptosis may have increased. Previous research indicates that increased PS exposure at the RBC membrane level could be involved in enhanced RBC adhesiveness to endothelial cells, but it does not appear to play a role in RBC aggregation [28,29]. However, RBC aging is accompanied by a decrease in sialic acid content [30], which could have resulted in increased RBC aggregability [31] and thus aggregation, in the MCC runners. Nevertheless, CD235-A expression, the main sialoglycoprotein of the RBC membrane, did not change in MCC runners. Sialic acids may be found in other glycoproteins, band 3, and some glycolipids [32]. Further studies are needed to understand the reasons for the increase in RBC aggregation during the MCC but not during the UTMB race. 

In contrast, blood viscosity measured at high shear rates decreased in UTMB runners after the race but remained unchanged in the MCC runners. RBC aggregates are dispersed at high shear rates [1]. This may explain why blood viscosity at high shear rates did not change following the MCC race. The decreased blood viscosity after the UTMB was mainly attributed to the decreased hematocrit. When blood viscosity decreases, vascular resistance is reduced. Therefore, the resulting decrease in vascular resistance would facilitate blood flow to the muscles [5]. This could help optimize athletes’ aerobic performance during long trail races that last for 20 to 45 h. In addition, the decrease in blood viscosity at high shear rates could result in decreased cardiovascular strain by limiting the cardiac afterload during exercise [2,5]. 

Exercise-induced dehydration generally results in a rise in hematocrit, with the degree of dehydration being related to the environmental temperature. However, our findings showed that less weight loss was associated with a greater decrease in hematocrit in both MCC and UTMB runners. Previous studies have reported plasma volume expansion [17,33,34,35] after long-distance running, resulting in hemodilution and decreased hematocrit after the race. Several factors could contribute to the increased plasma volume, such as an expansion of plasma albumin [17,36] or plasma sodium retention, as a result of increased aldosterone activity [33]. 

In the present study, the IL-6 level significantly increased after the UTMB, and the magnitude of hematocrit decrease was related to the magnitude of IL-6 increase when runners who completed the MCC and UTMB were pooled. IL-6 is a muscle-derived interleukin that is released into the circulation in response to exercise [37]. Inflammation is commonly suspected of promoting fluid shifts from the intravascular compartment, leading to tissue edema. This “relative hypovolemia” of the intravascular compartment would, in turn, promote water and salt retention, resulting in hypervolemia [17]. The consequences of increased IL-6 levels on the decrease in hematocrit is further supported by studies showing that the use of recombinant IL-6 in patients with cancer causes plasma volume retention [38,39].

The relatively small sample size of our study did not allow us to evaluate the impact of sex on the different parameters analyzed. Previous studies have reported sex differences in hematological parameters with higher RBC count, hematocrit, and hemoglobin concentration in males than in females [40,41]. Grau et al. [40] also described that RBC deformability was higher in females not taking hormonal contraception (HC) compared to women taking hormonal contraception and compared to males. Further studies are needed to test the sex differences on the hematological and RBC responses during such long-distance races. 

## 4. Materials and Methods 

### 4.1. Subjects and Protocol Design

Twenty-three endurance-trained runners participated in the present study after giving informed written consent. Eleven runners (5 males/6 females, 35.7 ± 9.0 yrs, 173 ± 8 cm, 65.4 ± 10.2 kg) participated in the MCC race and 12 runners (8 males/4 females, 38.0 ± 6.7 yrs, 171 ± 9 cm, 63.3 ± 8.9 kg) participated in the UTMB race. The course and weather conditions were similar between the two races. The mean daily temperature in Chamonix was 30 °C and 31 °C for the MCC and UTMB, respectively. The protocol was approved by the Ethics Committee (CPP Ouest VI, ethics committee agreement 19.03.14.41740 received on 05/02/2019), and the study was conducted in accordance with the Declaration of Helsinki. All subjects performed a maximal incremental treadmill test to determine their maximal oxygen consumption 4–8 weeks before the events. Blood was sampled in the medical center of the National School for Ski & Mountaineering (ENSA, Chamonix) before and 30 min after the races in EDTA and citrate tubes for hematological, hemorheological, spectrophotometric, and flow cytometry analyses. 

### 4.2. Maximal Incremental Treadmill Test

The maximal incremental exercise test was performed on a motorized treadmill (EF 1800, HF Tecmachine, Andrézieux-Bouthéon, France). The subjects started with a 4-min warm-up at 10 km/h. After 1 min of rest, the treadmill incline was set to 12%, and the test began at a speed of 5 km/h for women and 6 km/h for men. The slope was kept at 12%, and the speed was increased by 0.5 km/h every minute until the subject reached voluntary exhaustion. VO_2_max was determined by a breath-by-breath automated exercise metabolic system (Metamax, Cortex, Leipzig, Germany). 

### 4.3. Hematological and Hemorheological Analyses 

Blood viscosity was measured at 11.5, 45, and 225 s^−1^ after complete blood oxygenation and at native Hct using a cone/plate viscometer (Brookfield DVII+ with CPE40 spindle, Brookfield Engineering Labs, Natick, MA, USA). Hct was determined after blood microcentrifugation. MCV and MCHC were determined using a hematological analyzer (XN200, Sysmex, Villepinte, France). RBC deformability was assessed at 37 °C and 3 and 30 Pa by laser diffraction analysis (ektacytometry), using the Laser-assisted Optical Rotational Cell Analyzer (LORRCA MaxSis, RR Mechatronics, Hoorn, The Netherlands). The system has been described elsewhere in detail [42]. Briefly, 5 μL of EDTA blood was mixed with 1 mL polyvinylpyrrolidone (PVP; viscosity ≈ 30 cP) and sheared into a Couette system. A computer was used to analyze the diffraction pattern to determine an elongation index (EI), which represents RBC deformability. RBC aggregation was determined by light transmission (Myrenne aggregometer, Myrenne GmbH, Roetgen, Germany), at stasis after shearing at 600 s^−1^ [43] and after adjustment of the Hct to 40% with autologous plasma [42]. 

### 4.4. IL-6 and Markers of RBC Damages and Senescence 

Plasma free hemoglobin was determined by the measurement of hemoglobin absorbance at 576 nm using the Cripps method [44]. Blood samples were centrifuged for 10 min at 1000× *g*, and the supernatant, i.e., plasma, was harvested. Meanwhile, hemoglobin standards were prepared from 2.5 mg/dL to 100 mg/dL. The plasma and standard absorbance (Abs) were measured from 540 to 600 nm using a spectrophotometer (Synergy H1, Biotek, Winooski, VT, USA). For each sample, an Allen baseline correction was applied to limit background signals using the following formula: (2 × Abs 576 nm) − (Abs 560 nm + Abs 593 nm). A linear standard curve was created and used to determine the hemoglobin concentration (mg/dL) in the plasma samples. The percentage of hemolysis was determined using the values for plasma free hemoglobin, Hct, and total hemoglobin concentration, as follows: (100-Hct) × plasma free hemoglobin (g/L)/total hemoglobin (g/L).

Plasma MPs were quantified as previously described [9]. Blood samples were centrifuged at 1000× *g* for 10 min at 20 °C. The supernatant fraction was harvested and ultracentrifuged at 20,000× *g* for 20 min at 20 °C to isolate MPs. The supernatant was carefully discarded, and the pellet was washed twice in buffer 1 (10 mM HEPES, 136 mM NaCl, 5 mM KCl, 2 mM MgCl_2_, pH 7.4) containing 5 mM of EDTA for the first washing step and no EDTA (buffer 2) for the second one. MP pellets were resuspended in buffer 2 and stored at −80 °C until the day of analysis. RBC-MP content was evaluated by co-labeling with PS and CD235a. After thawing, MPs were diluted with staining buffer (10 mM HEPES, 3 mM CaCl_2_, pH 7.4). MP suspensions were co-incubated with Annexin-V-FITC (1/150 dilution, Beckman Coulter, IM3546) and anti-CD235a-PE antibody (1/2000 dilution, Miltenyi, 130-120-473) for 30 min in the dark at room temperature (RT). After incubation, samples were washed in staining buffer and analyzed by flow-cytometry for absolute MP quantification (Miltenyi Biotec, MACSQuant Analyzer 16, Bergisch Gladbach, Germany). The megamix kit was used to standardize MP acquisition gate based on fluorescent microbead size (0.5, 0.9, and 3 μm; Biocytex, 7803, Marseille, France) according to the supplier’s instructions. Unstained MPs were used to detect the samples’ auto-fluorescence, and single-color stained tubes were used for compensation settings. RBCs-MPs were defined as events that were both smaller than 1 μM and positively labeled with Annexin V-FITC and anti-CD235a-PE.

PS exposure on the outer membrane leaflet of RBCs was assessed using Annexin V-PE (Miltenyi, 130-118-363). RBC membrane CD47 and CD235a levels were measured by using anti-CD47-PE and anti-CD235a-PE antibodies, respectively (Miltenyi, 130-101-348 and 130-100-259, respectively). Intracellular reactive oxygen species (ROS) of RBCs were evaluated using 2ʹ,7ʹ-Dichlorofluorescin Diacetate (DCFDA, Sigma, D6883, Saint-Louis, MO, USA). Blood samples were centrifuged at 1000× *g* for 5 min at room temperature (RT), and the supernatant was discarded. RBC pellets were washed in PBS 1X (Corning, 21-040-CVR) and resuspended at 0.2% Hct in the appropriate staining buffer, according to the tested conditions. Annexin buffer 1X (Miltenyi, 130-092-820) was used as a staining buffer for PS exposure analysis, and PBS 1X was used for CD47, CD235a, and ROS levels. Depending on the conditions tested, RBCs suspensions were incubated with Annexin-V-PE (1:11 dilution), anti-CD47-PE (1:34 dilution), anti-CD235a-PE (1:400 dilution), or DCFDA (20 µM) for 20 min in the dark at RT. For the three markers, negative controls were prepared using unstained RBCs. Isotype control (1:51, REA-PE, Miltenyi, 130-113-438) was also used in CD47 and CD235a analysis to evaluate the antibody non-specific staining. After incubation, samples were washed twice in their respective staining buffer and analyzed by flow-cytometry (Miltenyi, MACSQuant Analyzer 16). For each sample, gating was performed on 100,000 RBCs per condition. 

Plasma IL-6 level was measured by Bio-Plex Multiplex immunoassay (Biorad, Hercules, CA, USA), using the Bio-Plex Pro™ Human Cytokine 17-plex Assay kit and the BioPlex 3D platform (Biorad), according to the manufacturer’s instructions.

### 4.5. Statistical Analyses

Data are expressed as mean ± SD. A two-way ANOVA with repeated measures (time × race), followed by an LSD post-hoc test when appropriate, was performed to compare the biological responses between the two races. Pearson correlation was used to test for the presence of associations between parameters. Chi^2^ analysis was used to compare gender distribution between the two groups. Statistical analyses were performed with GraphPad Prism 7 software (La Jolla, CA, USA). A *p*-value < 0.05 was considered as significant.

## 5. Conclusions

In conclusion, our study demonstrated that blood rheology behavior is different between a 40 km and a 171 km mountain race. Blood viscosity was impacted differently by the two races, with a rise noted after the 40-km, but a reduction after the 171-km. We suggest that inflammation, the decrease in hematocrit, release of RBC-MPs, and an increase in MCHC that occurred during the UTMB may explain the reduced RBC deformability and the decrease of blood viscosity at high shear rate. The low viscosity noted in UTMB runners could decrease vascular resistance, thereby facilitating blood flow to the muscles and optimizing aerobic performance.

## Abbreviations

Abs	Absorbance
CD235a	Glycophorin-A
EI	Elongation Index
Hct	Hematocrit
IL-6	Interleukin 6
MCC	Martigny-Combes à Chamonix
MCHC	Mean Corpuscular Hemoglobin Concentration
MCV	Mean Corpuscular Volume
MFI	Mean Fluorescence Intensity
MPs	Microparticles
PS	Phosphatidylserine
PVP	Polyvinylpyrrolidone
RBC	Red Blood Cell
RT	Room Temperature
ROS	Reactive Oxygen Species
UTMB	Ultra-Marathon of Mont Blanc
VO2 max	Maximum oxygen consumption
